# The CURE for the Typical Bioinformatics Classroom

**DOI:** 10.3389/fmicb.2020.01728

**Published:** 2020-08-12

**Authors:** Jennifer A. Bennett

**Affiliations:** ^1^Department of Biology and Earth Science, Otterbein University, Westerville, OH, United States; ^2^Biochemistry and Molecular Biology Program, Otterbein University, Westerville, OH, United States

**Keywords:** microbiology, bioinformatics, genomics, STEM, education, CURE, classroom

## Introduction

Bioinformatics is a field that combines biology and computer science to investigate relevant current topics such as annotation of the Human Genome Project and other genomes, protein structure and function, examination of disease processes and personalized medicine, evolutionary relationships and conservation genetics (as reviewed in Luscombe et al., 2001; Can, 2014). Today it is difficult to find a published article in biochemistry and molecular biology/microbiology that does not have a bioinformatics component. Thus, it is important for majors in the life sciences to have exposure to bioinformatics in the curriculum. A blended course format (Garrison and Kanuka, 2004) allows for short lectures and hands-on learning in the classroom combined with computer-based learning outside of the classroom in the form of literature searches, computer tutorials, and independent research that use information from the course applied to specific projects. The topics and techniques that students learn will help them navigate the vast amounts of information that are freely available in databases and inform them about how to manage that data and derive new information.

Bioinformatics is often a course taught in a workshop/computer lab format and in many instances a primarily lecture format. Adding a CURE, or Course-based Undergraduate Research Experience, has many advantages over traditional labs and lectures. CUREs have features of inquiry-based learning and also allow for participation in a larger project and community of researchers (Auchincloss et al., 2014). The Bioinformatics course described here incorporates many of the best teaching practices that have been called upon by numerous professional societies and are included in the 2011 Vision and Change Report (Brewer and Smith, 2011).

## Overview of the Blended Learning Workshop Format

My upper-level bioinformatics course was taught in the format of a workshop that was designed to keep students engaged both inside and outside of the classroom. At the beginning of each week I led the first workshop with what I referred to as a “Bio Byte,” a newsworthy current event in the field of bioinformatics that was often in the form of a short video clip. Each class incorporated a mini-lecture of ~10 min that described the bioinformatics tool(s) to be used that day, descriptions about when and why the particular tool should be used and the relevance to society. A guided demonstration ensued with students practicing the software and later applying it to their gene of interest, in a self-paced, hands-on experience. In the open lab format at the end of each class, students could practice the tools and converse with each other to gain additional knowledge in addition to asking for instructor feedback.

Beginning with student outcomes in mind, my Bioinformatics course was created using a backward design approach (Wiggins and McTighe, 2005). The desired outcomes of the course were for students to (1) Learn how to use various bioinformatics techniques, (2) Apply the techniques to answer pertinent research questions, (3) Explain how bioinformatics is connected to wet-lab experimentation, and (4) Generate and report novel data. The blended learning or hybrid format allowed for the majority of the semester to be taught in two 55-min sessions per week with additional time to work on the project and assignments that were posted to the blackboard learning site. It also allowed for some open computer lab days where students could work and ask questions. The atmosphere was relaxed for the students and fun to teach.

## A Project-Based Approach to Bioinformatics

With the intent of introducing large data sets and independent research, students were given access to RNA Sequencing data generated from my microbial genetics research program. The RNA-Seq experiment compared wild-type bacterial gene expression with that of a mutant under the same conditions (Bennett, unpublished). Students examined the data and chose a gene that was not already annotated in the spreadsheet. The job of each student for the rest of the semester was to characterize this chosen gene by conducting independent research and applying the various bioinformatics tools that they would be learning in the classroom. The fact that each student was assigned a different gene for investigation allowed a unique combination of both creativity and design-sharing within the classroom. Students were able and encouraged to help one another in an environment that promoted improvements to create better quality portfolios and more advanced analyses.

Instead of exams, each student was responsible for submitting a final portfolio showing mastery of the various bioinformatics tools that they learned in class as applied to their specific gene. The portfolio was the culminating project for the bioinformatics course, and required figures and corresponding legends in the format of a publication along with annotations about the techniques used. A minimum set of expectations was given for the gene analysis portfolio, however students had the freedom to characterize their gene in ways that extended beyond these expectations. The result was multiple student portfolios that showcased additional tools not discussed in class and advanced functions of the tools that we had covered.

## Long Term Positive Outcomes

The portfolio specifically allowed students to demonstrate their ability to apply each bioinformatics tool to their chosen gene of interest. The independent research project and resulting portfolio could be listed on student applications, curricula vitae, and resumes. Additionally, each of the 12 students in the class was required to give a short oral presentation, describing what they had learned about their gene and its possible role in the cell. Three of the students also seized the opportunity to present their bioinformatics research, and their abstracts were accepted for poster presentation at the Ohio Branch Meeting of the American Society for Microbiology. Two of the students decided to pursue Ph.D. programs in bioinformatics/computational biology, in large part crediting the experience that they had in the bioinformatics course. Two additional students also entered Ph.D. programs bringing with them bioinformatics knowledge they learned in the course that will be extremely useful to their dissertation research. There was one sophomore student in the spring 2018 course, who is about to graduate and is currently applying to Ph.D. programs where she hopes to combine her skills in bioinformatics and microbiology. Spring 2018 was the second time that I taught Bioinformatics. I first taught the course as an experimental course in Fall 2013 with only six students enrolled. During the first iteration of the course, it was taught in a very similar workshop format with a final portfolio, only without the exposure to the RNA-Seq dataset. Students chose a gene of interest that was uncharacterized from the *Streptomyces* genome (Bentley et al., 2002) and presented on that gene. One of the initial six students chose to enter a graduate program to pursue bioinformatics research based on his bioinformatics experience at Otterbein and is about to graduate with a Ph.D. in Biochemistry with dissertation research entirely in the area of bioinformatics.

The features that make the guided workshop approach with a novel independent research project so successful are hands-on direct application, student ownership of an important project, the ability of students to customize their portfolio and pursue advanced topics, and the ability to communicate their data in both written and oral form. Wilson Sayres et al. (2018) published a set of bioinformatics core competencies in 2018 that are readily achieved in the framework of the bioinformatics course described here. The students must read and evaluate the primary literature and directly apply bioinformatics techniques. Their final product is a source of pride. They produce data that has the possibility of publication and they are part of a larger community of researchers within their classroom and in the field of microbial genetics. The data continues to make an impact as it influences future studies in my research program.

Concepts and techniques that the students learned and applied in the Bioinformatics course included BLAST, multiple sequence alignments (Clustal), phylogenetics, domain mapping (SMART and Pfam), analyses of protein-protein interactions, and protein modeling (RaptorX, Cn3D, and pyMOL). Some of the proteins were also 3D printed in collaboration with engineering students at The Point, Otterbein's STEAM innovation center. Connections to wet-lab experimentation and other disciplines were introduced throughout the semester, including the next steps in the analysis pipeline. For example, after using bioinformatics to identify and begin the characterization of novel genes, some of the gene expression data obtained through RNA-Seq could be verified using Real Time PCR. Genes could be deleted using such tools as the Lambda Red System (Datsenko and Wanner, 2000) or CRISPR-Cas9 (Wang et al., 2016) to determine the mutant phenotype and thus provide experimental evidence for the role of the gene as compared to that predicted using bioinformatics.

The following examples illustrate the long-term impact of bioinformatics skills learned by undergraduates on my research program. Undergraduate research students in my lab have successfully completed Real Time PCR experiments for two genes identified in the RNA-Seq experiments introduced into the bioinformatics class, and a student is currently using the Lambda Red System to delete a gene of interest identified in the bioinformatics course. Another gene identified in our RNA-seq experiment was fortuitously disrupted in a transposon experiment and we are studying it and other similar genes identified using bioinformatics in light of the RNA-Seq data. Hull et al. (2012) serves as a past example of undergraduate co-authors contributing significant research to a published paper using bioinformatic analyses. One student co-author contributed entirely through bioinformatics research, stemming from a portfolio completed as an independent study in my lab. This is the format that I have continued to employ in my bioinformatics course. The published paper incorporates the following skills performed by undergraduate co-authors: construction of genetic maps, primer design, sequence analysis, BLAST to identify similar genes in the genome and to identify orthologues in other species, sequence alignments, and protein domain mapping. As part of my bioinformatics course, students employ these skills that our lab typically uses to present and publish, in addition to many more techniques such as those listed above.

## Discussion of Educational Applications

Bioinformatics is a course that lends itself especially well to a blended course and workshop format. The application of techniques to a novel gene of interest in a progressive order kept students engaged with a sense of strong ownership. Only a computer lab or student laptops are required. My entire course made use of databases and software that were freely available to the public. The bioinformatics tools were easily accessible and relatively simple to learn for instructors with little bioinformatics background because the programs employed Graphical User Interfaces (GUIs) that do not require programming knowledge. However, all of these exercises can be easily extended to include introductory scripting for students. The introduction of some command lines into the course is advantageous for students to better understand how their data is being obtained.

The course format used in the bioinformatics course described here can be transferred to other portions of the biology and microbiology curricula. A small portion of a course can be devoted to a bioinformatics analysis of research data using any of the techniques from the full bioinformatics course, allowing students to make important contributions to large projects. The course used a bacterial genome of interest to my research program, but the same techniques can be applied to any organism, based on the interests of the instructor. In my course, I introduced RNA-seq data, but we also have a proteomics project where we used mass spectroscopy to identify binding proteins discovered in bead capture experiments. Students could easily have investigated these proteins instead. Any bioinformatics course or module could also include a functional genomics component where students are involved in complimentary wet-lab experimentation along with the bioinformatics analyses. In summary, this type of bioinformatics CURE can readily be used in full courses or modified for modules within a course to actively engage students in meaningful research with high learning gains.

## Author Contributions

The author confirms being the sole contributor of this work and has approved it for publication.

## Conflict of Interest

The author declares that the research was conducted in the absence of any commercial or financial relationships that could be construed as a potential conflict of interest.
